# Midwives’ strategies for coping with barriers to providing quality maternal and neonatal care: a Glaserian grounded theory study

**DOI:** 10.1186/s12913-021-07049-0

**Published:** 2021-11-03

**Authors:** Yakubu Ismaila, Sara Bayes, Sadie Geraghty

**Affiliations:** School of Nursing and Midwifery, Edith Cowan University, 270 Joondalup Drive, Joondalup, WA 6027 Australia

**Keywords:** Midwives, Maternal health, Neonatal health, Quality care, Psychological resilience, Coping, Ghana, Low and middle-income countries, Developing countries

## Abstract

**Background:**

Midwives face direct and indirect barriers in their workplaces that have negative consequences on their ability to provide quality care to women and neonates, however, they still carry on with their duties. This study aimed at investigating the coping strategies that Ghanaian midwives adopt to be able to complete their work.

**Methods:**

Glaserian Grounded theory was used in this study. Data were collected through non-participant observations and semi-structured interviews. The study participants included 29 midwives who worked in labour/birthing environments and a pharmacist, a social worker, a national Health Insurance Scheme manager and a health services manager.

**Findings:**

The midwives’ motivation due to their strong desire to save the lives of women and neonates and their strong affection for the midwifery profession was identified to help them cope with the barriers that they faced in their workplaces. The midwives’ motivation was found to spur actions such as improvising, taking control of the birthing process and the birthing environment and the maintenance of social and professional networks to help them to complete their duties.

**Conclusion:**

Ghanaian midwives face myriad barriers in their workplaces; however, they are able to adopt coping strategies that enable them to complete their duties. The provision of care resources for maternity services in the country will reduce the barriers that the midwives face and improve the quality of maternal and neonatal care. In the short term however, pre and post midwifery educational programmes should focus on developing resilience in the midwifery workforce to help midwives cope more effectively with the challenges that they face in their workplaces.

**Supplementary Information:**

The online version contains supplementary material available at 10.1186/s12913-021-07049-0.

## Background

Maternal and neonatal mortality is very high in low and middle-income countries (LMIC), despite global efforts towards its reduction. It is estimated that 99% of all maternal deaths occur in these settings [1]. The midwifery model of care, that focusses on using well-educated and qualified midwives for the provision of maternity care, has been shown to not only reduce maternal and neonatal mortality but to also have a positive impact on education and economic empowerment [2]. However, of the estimated global shortfall of human resources for health, it is estimated that midwives and nurses account for half [3].

The International Confederation of Midwives defines a midwife as a person who has been registered/and or legally licensed to practice midwifery after successfully completing the stipulated course of studies in a midwifery education programme that is recognised in the country in which it is located [4]. In recent years, high demands on midwives, due to their increasing workload because of a rise in birth rates and a growth of the complexity of pregnancy and birth care, has become a source of pressure on them, thereby affecting their efforts to provide quality care to women and neonates [5, 6]. Additionally, midwives are facing professional, social, and economic barriers such as absence from policy dialogue, insufficient supplies and equipment, lack of recognition of skills, lack of support for housing and transport, lack of safety and security, and sex inequality [7–9]. Barriers to midwives’ ability to provide quality care to women and neonates have physical, psychological, and socioeconomic consequences on them. Psychological consequences of midwives’ ability to provide quality care include fear, anxiety, anger, and sadness as well as stress, desperation, insecurity, and demotivation [10–13]. Reported physical consequences on midwives’ ability to provide quality care include extreme exhaustion and fatigue and physical attacks as well as ill-health and other medical challenges such as infection risks [14–16]. Socioeconomic consequences to barriers to midwifery work include pressure on their marriages as well as on their social networks, inadequate salaries, and other financial effects that result from providing care to women and neonates from low and irregular income [7, 17]. All these consequences lead midwives to disengage emotionally from their work, which has a direct impact on outcomes for women and neonates [5]. The consequences of the barriers to the ability of midwives to provide quality care have been found to result in burnout and moral distress which can have negative effects on their retention [5, 7, 18].

Many midwives are, however, thriving in the profession due to their ability to devise resilience strategies that help them cope with the adversities of the profession [6]. Resilience can be defined as a dynamic process whereby individuals or groups adopt positive adaptation within the context of significant adversity [19]. Positive adaptation usually occurs using coping strategies that are devised by the individuals or groups [20].

Midwives have been shown to employ adaptation strategies to cope with the barriers and the effects of those barriers in their working lives [6, 21, 22]. An important coping strategy that is commonly adopted by midwives is trying to control what they can in their work environment for them to be able to work effectively [21]. This process may involve the use of strategies such as assertiveness and other confidence boosting strategies such as self-affirmation and the insistence on their job autonomy when required [3, 23]. Social and professional support from work colleagues and supervisors is another factor that help midwives cope with the barriers in their job environment [5, 22]. Apart from work colleagues and supervisors, support from family and friends has also been identified to help midwives’ coping abilities [21, 23]. In addition to the above-mentioned factors, engaging in sports and other hobbies, having a work life balance and therapeutic activities outside the workplace is believed to help midwives and other health workers’ ability to cope with stress and or distress [6, 21, 24]. Midwives’ love of the profession, their sense of purpose, the fulfillment that they get from performing their duties, and their acknowledgement of the expectations that patients or families have on them are other coping factors that has been found to help midwives thrive [25, 26].

As a lower middle-income country, Ghana’s maternal and neonatal mortality of 310 per 100,000 and 25 per 100 live births respectively are both high and this is attributed to poor quality maternity services [27–29]. One measure adopted to improve outcomes for women and neonates is the improvement of skilled attendance at birth by the training and recruitment of more midwives, but the country still faces a shortage of midwives [9, 30]. This shortage combined with other barriers such as inadequate essential equipment and supplies, inadequate infrastructure and referral resources, low incentivisation and insufficient in-service training and the physical, psychological and social effects of these barriers negatively affect the ability of midwives to provide quality care to women and neonates [8, 9, 31, 32]. Nonetheless, the midwives seem to be coping with these challenges to still provide care to women and neonates [9, 12, 33]. Currently, only a few studies have focused on how Ghanaian midwives cope with the challenges in their work. This study therefore aims at examining the coping strategies that the midwives adopt to be able to carry out their duties in the face of their workplace barriers and the effects of those barriers. Understanding how midwives cope with the challenges in their work could provide useful information to guide the support and training of not only Ghanaian midwives but also other midwives who work in similar contexts.

## Methods

Glaserian (classic) grounded theory (GT) methodology was employed in this study [34]. The methodology was chosen for this study because it provides a way to explain ongoing behaviours of participants and the way they solve issues of concern [35]. The first author (YI) collected all the data for the study and led the analysis. In Glaserian GT it is important that the researcher keeps theoretical sensitivity low at the beginning of the study to allow the data analysis to augment it, therefore, it is recommended that the researcher discerns and purges his or her prior assumptions on the phenomenon under study as much as possible at the inception of the study [34]. Accordingly, similar to what happens in bracketing, YI noted all prior assumptions he had as someone with direct and vicarious personal and professional experience of the Ghanaian maternity system and discussed it with SB and SG [36]. The participants’ narratives were thus heard and analysed objectively without being obscured by any prior assumptions.

### Participants and setting

The study took place in the Greater Accra Region in southern Ghana. A large proportion (90%) of the population in this region reside in urban and peri urban areas, however a considerable number of the population lives in rural areas [37]. The region has been identified to have the highest number of public health facilities in the country [37]. To practice as a midwife in Ghana persons must enroll in a midwifery college for a two years or three years course for a certificate or diploma in midwifery respectively or obtain a bachelor’s degree in midwifery from an accredited university [38]. Ghana practices a midwife led maternity system [39]. Midwives practice autonomously, only referring to obstetricians in the event of complications that are beyond their competencies. The midwifery profession in Ghana is female dominated with only a few practicing male midwives.

Midwives who work in the labour wards in Ghana Health Service (GHS) facilities were the main target population for this study. One midwife was however recruited from a private hospital to serve as a negative case. To be recruited into the study the midwives must have worked in the labour ward/birthing environment for at least one year. Midwives were recruited from 10 health facilities in seven districts of the study region - four health facilities from urban areas, three health facilities from peri-urban areas and three from rural areas. Purposive and theoretical sampling were used in this study. At the inception of the study three facilities were purposively selected – one facility each from the metropolitan areas, the peri urban areas and the rural areas. Purposive sampling was also used in the recruitment of the interviewed participants. The study participants were recruited by the first author in their work environments. As categories emerged, during the data analysis, theoretical sampling was then used to recruit participants who could provide data to solidify the categories and to find the relationships between them. A pharmacist, a social worker, a National Health Insurance Scheme manager, and a GHS management staff were recruited during theoretical sampling. These participants were recruited because their work impacted the functioning of the midwives.

### Data collection and analysis

Field work for the study was conducted between January and August 2018. Data were collected through non-participant observations and semi-structured interviews. The non-participant observations were carried out before the interviews and focused on the midwives immediate working environment including infrastructure, water and sanitation, equipment and supplies, and protocols. During the initial stages of the study, midwives were approached by YI for interviews after their shifts, during the non-participant observations. Face-to-Face interviews were conducted with the participants using an interview guide that was developed by the authors (see additional file 1). Revisions were made to the interview guide as the study progressed. The interviews that lasted between 45 and 60 min were conducted in English language in places chosen by the respondents that afforded them privacy and confidentiality. The initial interview questions that are relevant to this article included the following: What are the barriers to your ability to provide quality midwifery care to women and neonates? Can you explain to me how these difficulties affect your ability to provide quality care? What do you do to reduce the effects of these barriers?

The incidents, codes and categories that were arrived at from the analysis of non-participant observation data guided the interviewer’s probes during interviews. All the interviews were audio recorded with the participants’ consent. The first author listened to the interviews and transcribed them verbatim. Data analysis was carried out using open and theoretical coding. Memos were made by the researcher as the data were analysed. Open coding involved the identification of important words or groups of words and phrases and labelling (‘coding’) them. The codes, including those acquired through non-participant observations were constantly compared, and similar codes were grouped together and delimited into a core category and three subcategories and their properties. During theoretical coding the relationships between the core category and the subcategories were established.

### Ethical consideration

The study received ethical approval from the Human Research Ethics Committee of Edith Cowan University, Australia, (number 18162) as well as the Ethical Board of GHS (GHS-ERC: 009/10/17). During recruitment, all participants were provided information sheets. Participants were informed that they could leave the study or stop interviews at any time without any consequences to them. All the participants in the study signed consent forms when they were recruited. Although the research was deemed to be of low risk to the participants, a professional counsellor was available to provide support to participants if they felt distressed by recounting negative practice experiences (none required the service). The first author (YI) arranged with the second and third authors (SB and SG), who are experienced midwives, for debriefing in case he heard about or witnessed difficult or distressing practice situations. To ensure the confidentiality of the study participants, all the names that are used in the findings section are pseudonyms.

### Trustworthiness

The study was overseen by SB ang SG who are experienced Grounded Theorists. To ensure the dependability of the study results, the first 45% of the interviews were coded and categorised by at least two members of the research team. Further, the categories and the sub-categories were always agreed on by all the authors through discussion. The gathering of data through non-participant observation and interviews and the interviewing of three non-midwife participants enabled the acquisition of different slices of data that were constantly compared to arrive at the study results. This is similar to method and data source triangulation and contributed to the studies rigour. During theoretical coding, a midwife who works at a private health facility was interviewed to serve as a negative case. Data from her interview totally contrasted those acquired from the other midwives thereby proving the validity of the study results. As the Findings of GT studies are arrived at by building on initially discovered codes and categories, some of the midwives were interviewed more than once. For those midwives, the codes and categories that were acquired from the analysis of their previous interviews were presented to them for clarification before the subsequent interviews, similar to what happens in member checking. Also, at the final stages of the research, the results of the study were presented to midwives from an adjourning region (Eastern Region) during one of their regional meetings for critical review, feedback or revision to confirm the fit, generality, and understandability of the findings.

## Results

The study participants comprised 29 midwives, aged between 26 and 59 years and a pharmacist, a social worker, a National Health Insurance scheme manager, and a health services manager. The average years of experience of the midwives was eight years. The demographic information of the midwives in the study are provided in Table 1.

**Table 1:** Demographic characteristics of midwife participants

Demographic variable	Category	Frequency
Age	25–29 years	4
	30–35 years	12
	36–39 years	3
	40–45 years	3
	46–49 years	2
	56–59 years	5
Years of experience	1–5 years	12
	6–10 years	8
	11–15 years	6
	16–20 years	3
Education	Diploma in midwifery	25
	Bachelor of midwifery degree	4
Health Facility type	District Hospital	22
	Health Centre	7
Health facility location	Urban	16
	Peri-urban	7
	Rural	6
Sex	Female	28
	Male	1

The results of the study indicate that despite the barriers that the midwives faced and the consequences of those barriers, they adopt coping strategies that enabled them to complete their work. One core category – ‘being motivated’ and three sub-categories namely, ‘improvising’, ‘being one step ahead’, and ‘maintaining a support network’ were found to enable the midwives cope with the barriers that they face in their workplaces. Table 2 provides a highlight of how the category and sub-categories were arrived at.

**Table 2:** Examples of midwives’ expressions, their substantive codes and the category or sub-categories

Original quote	Substantive codes	Category/sub-category
“Seeing the mothers alive with their babies … that is the greatest motivation I have”.	Being motivated by the desire to save lives	Being motivated
“it is my contribution to my community and my society”.	Being motivated by the affection for the profession	
“We don’t have a [number] lot of equipment to work with. Every day we improvise”.	Improvising in the face of insufficient equipment	Improvising
“We tear a pillowcase to receive the baby to prevent the baby from chilling”.	Improvising in the face of insufficient supplies	
“if you do not keep an eye on the client, by the time you realise, [something is wrong]. Maybe the heart rate is gone”.	Pre-emptive action to prevent negative outcomes	Being one step ahead
“We [the midwife and the woman] will go first, then you [client’s family or accompanying persons] will follow”.	Proactive action to avoid negative outcomes	
“I contact my more experienced midwives. They support”.	Support from colleagues	Maintaining a support network
“[Your husband may say], “Ok, it will be well. Let’s hope for the best”.	Family support	

### Being motivated

Despite the barriers and the consequences of the barriers that the midwives faced in their work, they  were found to be coping and still providing care to women and neonates due to their motivation. The midwives indicated that their greatest source of motivation for continuing in their profession despite the challenges is their strong desire to save the lives of women and neonates and their strong affection for the midwifery profession. The midwives indicated that they were motivated by their strong desire to enable women have safe births and healthy babies, as women come to them because they trust this is possible. This is represented by Fareeda in the following quote:

“*Seeing the mothers alive with their babies. For me, that is the greatest motivation I have had. A mother will come since she can’t deliver in the house. She has the motive that “when I come to the hospital I will come and meet someone good”, that is my pride.”*

The midwives’ motivation is also underpinned by the intense affection that they have for the midwifery profession. The midwives reported that they saw their profession as a calling through which they can contribute to their societies as indicated by Mariama below:

*“One thing which motivates me to be a midwife is that it is my contribution to my community and my society. The only way that I can do it (contribute to society) and I know people will appreciate it is to be a midwife. Because, I will have to help the pregnant woman from antenatal through delivery. I always feel happy when I do that. That is my motivation. Not even the salary”*

As a result of their strong affection for the midwifery profession, the midwives shared that they get fulfilled when they take care of women and neonates, for instance as indicated by Dela in the quote below:

*“I feel fulfilled when a pregnant woman walks in and even if it is a complicated case, we get through it and mother is alive and baby is doing well. I get some gratification from that, and I think that is motivating enough to keep me going.”*

Further, the midwives reported that, because of their affection for the profession they “*keep going”* (Elsie) even in the face of the heavy workload that they face. Others talked about upholding the best standard of care possible even though they receive inadequate financial compensation for doing so. Akos, for example, said:

*“As I am sitting here, I don’t have money [I’m not rich] but it does not affect the way I deliver. Even my salary is not enough, but I still deliver*.”

The midwives shared that they always want to give their best as this gives them a sense of pride. One example is in Ayele’s quote below:

*“Do your best so that when you go home, and they say “ayekoo” [well done] you can respond “yaaye” [thank you]. “Yaaye” means that you have done well. So that when you go home, and your husband greets you and ask you “how is work?” you will not feel guilty.”*

The midwives in this study indicated that because of their motivation they were spurred to adopt other coping strategies that were captured in the following subcategories: 'improvising', 'being one step ahead' and 'maintaining a support network'.

### Improvising

The midwives reported that their motivation spurred them to improvise as a way of coping with the barriers that they faced in their work. Respondents indicated that as part of their training they are always encouraged to improvise when they are faced with challenging clinical situations. Buruwaa spoke for the group when she shared that they *“don’t have a lot of [a number of] equipment to work with”*, and this necessitates that they improvise by making do with whatever equipment is available to enable positive maternal and neonatal outcomes. This was expatiated further by Mercy in the following example:

*“It is only the labour ward which has a flow meter, so if theater recovery have emergency and they come for it and later we get an emergency, we can’t use it on the patient who is critical. So what can we do? We use an ambubag instead of face and mask.”*

The midwives, as exemplified by Mansah, stated that they “*wish[ed] that the things [equipment] are there so that we [they] can do our [their] best”.* However, she went on to indicate that “*when something [equipment] is not there”,* they are forced to do the next best thing or use what they can to get the best outcome. For example, Nakie indicated that “*if there is no ambubag, you can cover the baby’s mouth with gauze and breathe into the baby.”*

The respondents indicated the occurrence of clinical areas borrowing or sharing as a way of improvising to combat the effects of insufficient equipment. For instance, Clara, in the quote below, said.

*“The equipment, we borrow from other wards. For instance, the BP apparatus, we borrow from [another] ward. When the oxygen is finished, we go for the one at the [operating] theater.”*

Other modes of improvisation mentioned by midwives included sterilising equipment with bleach instead of autoclaving when birthing equipment is immediately required following use to attend to more women, with time being a crucial factor. Some midwives reported not having autoclaves in their health facilities, where the participants working in these facilities described that they always had to sterilize their equipment with bleach. Furthermore, some midwives indicated that they *“cut [the umbilical cord] with [a] blade”* (Ayele) when there were no more sterile scissors available.

Respondents’ capacity to improvise was also reported to extend to situations where women present in labour without the necessary items for their birthing. They went on to explain that, when this happens, they take the initiative to *“collect some [birthing items] from other clients”* (Selasie*)* who have spare items. According to respondents, when they are unable to get what the woman needs from others, they improvise further. This is indicated by Nakie in the quote below, where she refers to when women arrive without wraps for their neonates and she is unable to obtain any from other women:

*“Some women come to deliver without cot sheet (baby wraps) or even simple things they will use for the baby. We tear a pillowcase to receive the baby to prevent the baby from chilling.”*

Further complicating the challenges that some of the participants faced that have been described, the midwives also face the day-to-day uncertainty of whether there will be electricity where they are working. True to their adaptability though, even this reportedly did not prevent midwives from doing their job. In the face of frequent power outages and the resulting insufficient lighting, respondents indicated that they improvised by using the flashlights/torches on their mobile devices. For example, Sika, like the others, said, she just uses her “*… phone light to do the delivery.”*

### Being one step ahead

The participants indicated that to achieve positive outcomes for women and neonates despite the extensive challenges that they faced, they felt the need to be ‘one step ahead’ in the care-giving process through close and cautious monitoring of those in their care. In a care environment where client numbers are high, the resources for monitoring the progress of labour are insufficient, and referral is challenging, the midwives indicated that they still must timely preempt any deviation from the normal labour progression, not necessarily sticking to the usual monitoring time intervals. They did this by, as Kafui describes, *“keeping an eye on the person”.* This is necessary, as indicated by Mansah, because “*if you do not keep an eye on the client, by the time you realise, [something is wrong]. Maybe the heart rate is gone.”*

This proactive coping strategy of maintaining vigilant monitoring of women and neonates was indicated by midwives to also include making sure that their medical colleagues have enough information that is needed to act when things deviate from normal. Kafui further explains in the statement below, of the chaos caused when midwives are not well-informed about the women they cared for:

*“If you know your stuff, that is it. Because you’ve called him that there is an emergency, when he asks you what you have done and you can’t say, the doctor will feel you don’t know what you’re about.”*

These initiatives of the midwives’ amount to trying to exercise control over care situations and their environment when there is a lot that is unpredictable. Another way in which midwives did this was by prevailing upon their colleagues to fulfil their obligations and by taking control of risky situations to minimise negative outcomes. For instance, the rural midwives indicated that being proactive when dealing with high-risk situations enabled them to ensure prompt referral for women to facilities better equipped to care for them, even when women or their support persons are not motivated to accept the recommendation.

*“Sometimes when they come [and they are referred elsewhere], they will say they are going home [to prepare] before [they travel]. Especially when they are bent on not going. But some of these cases, it is not a matter of going home. You came from home, where you could not [manage], instead of going [to the referral facility], you say you are going home to collect money or to collect something. We [the midwife and the woman] will go first, then you [client’s family or accompanying persons] will follow. So that one, you have to take immediate action. I insist that the right thing is done. I don’t joke.”*

### Maintaining a support network

It is well known that the work of midwives can result in enormous pressure, which can cause distress to them. Midwives in this study indicated that because of their motivation, they maintain a support network that keeps them functioning in the face of the barriers that they faced. It was found that support was commonly obtained from three sources: colleagues, family, and faith. The midwives were found to have a strong sense of duty towards their colleagues. Mansah, for instance, indicated that *“it is my duty and if I do not go [to work], nobody will. Someone has come in the morning and her shift has ended so if you do not come … [she cannot go]”.*

The respondents indicated the value of the support of their clinical colleagues when they faced challenging cases and when they got distressed due to negative clinical outcomes. It was unearthed in this study that midwives call upon their clinical colleagues for support during emergencies. In facilities that have onsite accommodation, midwives can call on their colleagues to come out of their homes to help them, where midwives rarely object, as indicated by Fareeda: *Since we stay around, in case of difficulties, you can call your colleagues to come and help.* The respondents indicated that when they have negative outcomes, “*having people around” (kafui)* to talk to helped them cope with their distress*.* Usually, the midwives talk to their colleague as a first point of contact to help them cope with the negative effects of difficult practice experiences. Kafui gave the following example:

*“Just before Christmas I had two still births, so I talked to her [a midwife colleague] and another girl, Vivian. I usually come for night [shifts] so we decided that I break from night and come for day [shifts] for some time.”*

The midwives’ support networks also extended to colleagues in different facilities, especially when there is the need to refer women or neonates. The respondents noted the vital importance of mobile phones in helping them maintain contact with their colleagues and other midwifery staff in other health facilities especially for the management of emergencies. This is further expatiated on by Ashokor in the following quote:

*“These days, through our efforts, some of the doctors have given us [phone] numbers. When we are in difficulties, we call. And they direct you what you should do … . They are also very busy, so at times I contact my more experienced midwives. They support. They will tell you, do this and that.”*

The respondents indicated that there is a simple mHealth innovation that connect most of the health facilities in the region whereby they post their emergency cases on a mobile platform (Kybele mobile platform) and follow up with calls to get advice and help from their colleagues, as indicated by Margaret below:

*“You have to call [and someone will respond] “‘please, Ridge [hospital] is full, Achimota [hospital], can you help? Mamoobi [hospital], can you help? [Then] if they get somebody [health facility] they will call you and say, “send the case to [let’s say] Ridge” [hospital]” Ambulance driver [National Ambulance Service] is also on it.”* Midwives also indicated that their immediate families are a source of support that helped them cope with the distresses in their job, as indicated by Elsie below:

“*At times you will leave here crying because someone has uttered a word to you but when you get home and you meet your family, your kids, you forget about that and now your thought is with them. So, you look at them and you tell yourself, life must go on. Your husband too might ask you: “sweetie how is work”? [you may say] “Today when we went this and this and that”. [Your husband may say], “Ok, it will be well. Let’s hope for the best”.*

The midwives’ motivation was also found to spur them to seek the intervention of God via their spiritual connection through faith in their day-to-day duty of birthing and taking care of women and neonates. They indicated that praying when they faced challenging situations helped; Nakie stated:

*“You keep praying. We are human beings, and we know God does everything so we pray that even if something [should happen] at all, God should intervene. Prayers also works, and God does it.”*

Another respondent, Fafa also indicated that prayers was one of her ‘preventive’ coping strategies: *“I pray and commit my hands, my eyes, and my movements in God’s hands before I touch a patient.”*

## Discussion

Ghanaian midwives are striving to provide quality care for women and neonates to help reduce the countries high maternal and neonatal mortality. However, low midwife numbers together with other challenges such as inadequate essential equipment, supplies, and infrastructure, poor referral resources and negative client attitudes adversely affect their efforts to ensure positive outcomes for women and neonates [9]. Nonetheless, the midwives in the study context adopted coping strategies to enable them to still get good outcomes for women and neonates because of their strong motivation. The midwives’ motivation, that was identified to be underpinned by their strong desire to save the lives of women and neonates and their strong affection for the midwifery profession was found to help them cope with the barriers that they face in their work. In a study in Mozambique by Adolphson, Axemo and Högberg [40] midwives were shown to derive motivation from their ability to save lives, provide hope to their clients and from the sense of feeling that their work was beneficial to others. This finding is also in consonance with the findings of Bloxsome, Bayes and Ireson [41] and Cope, Jones and Hendricks [25], who indicated in their studies in Australia that their participants thrived in their profession because of their love for the profession and the value that they place in their role. Petrites et al. [26], in a study to find out how midwives and obstetricians cope with high perinatal deaths found that they were motivated by their sense of purpose and the sense of awareness of patients’ pain and suffering.

In the face of lack of equipment and supplies, the midwives in this study admitted that they are driven by their strong motivation to improvise by borrowing and sharing equipment or supplies, using alternative equipment that can produce positive results, and performing actions or using materials that can ensure positive outcomes. In another study in Ghana that investigated the perception of health workers on the quality of neonatal care, the respondents were also found to be improvising by doing direct mouth-to-mouth resuscitation on neonates when the required equipment was not available, as well as using improvised tools, akin to the midwives in this study’s use of their mobile phone flashlight to provide or augment lighting during birthing processes [42]. Although the midwives seem to be achieving positive outcomes through their improvisation, the long-term effects of this strategy on care quality and overall outcomes need to be investigated.

Studies in midwife resilience have indicated that midwives’ ability to control their environment is a coping strategy that enables them to thrive in their work [21, 23]. In the sub-category labelled ‘being one step ahead’, the midwives’ ability to take control of the processes of care are evident through close and careful monitoring of women, maintaining effective communication with medical doctors, and their assertiveness in ensuring that their colleagues and women complied regarding actions that favor positive outcomes. Jacobson and colleagues [43], in a study conducted in the USA to understand how interdisciplinary communication affect patient safety, underscored the importance of effective communication between midwives and other clinical staff in ensuring positive outcomes for birthing women. Adolphson, Axemo, and Högberg [40] also identified good collaboration and teamwork by midwives and other midwifery staff as an important factor that enabled the provision of quality care to women and neonates. In a study in Tanzania by Tibandebage et al. [11] it was shown that midwives’ assertiveness ensured that their colleagues provided prompt care to birthing women. Schack, et al. [13] also outlined the importance that midwives attribute to controlling the care environment: their participants described, for example, preparing equipment beforehand and checking on the functioning and availability of equipment. Pre-planning for any eventuality was also indicated by Hunter and Warren [23] as an adversity coping strategy used by midwives.

The findings of this study indicate that midwives rely on their colleagues for support when they have challenging cases as well as when they are distressed due to negative outcomes. Support from relationships both in and outside the workplace has been found to help midwives cope with the challenges in their work [3, 6, 21]. Geraghty, Speelman, and Bayes [5] in a study to examine the implications of work-related stress on midwives identified that when midwives discuss their challenges with likeminded colleagues it has an ameliorating effect on their distress. Geraghty and colleagues [5] indicated that midwives who work in the same environment form mutual support groups to help them cope with the negative effects of their job environment. The participants in Geraghty and team’s study used the phrase ‘having each other’s back’ (p.301) to emphasize the informal mutual support that midwives offer one another to be able to carry on with their work.

The finding in this study is also in congruence with the finding by Hunter and Warren [23] when they investigated resilience in midwifery that, talking to colleagues gives midwives perspective on the challenges that they encounter in their workplaces. According to Wahlberg, Högberg and Emmelin [3], who explored the processes that Swedish midwives go through when they experience severe events in the maternity, when individuals who have been through a certain experience provide support to colleagues, it boosts the confidence of the person from whom help is sought. This process has a two-way benefit. Therefore, this study’s finding that the midwives depended on their colleagues for support is advantageous as it will help boost coping abilities among the midwives. It will therefore be beneficial if colleague mentoring is instituted in the midwifery profession in Ghana because, the nurturing of confidence in personal coping abilities has been identified as a trait that is learnt over time [23]. Dartey, Phetlhu and Phuma-Gaiyaye [44] have indicated the absence of institutional measures to help midwives to cope with maternal deaths in the country. Calling in off-duty colleagues to support during difficult situations should, however, be looked at, as it could be a source of additional stress to the already overworked midwives.

Although Halperin and colleagues [22] who studied how midwives cope with stressful clinical life-threatening childbirth situations emphasised the empowering effect to midwives of support from supervisors, the midwives in this study did not mention receiving support from their supervisors; they only spoke of support from their peers. Further investigation should therefore be carried out on this phenomenon so that, if necessary, measures could be put in place in the labour wards to improve supportive supervision to empower the midwives to enable them cope with the challenges that they face.

Support by people who are external to the midwives’ workplace, such as that provided by family and friends, has been indicated by others to help increase midwives’ resilience [6, 21, 24]. Apart from their work colleagues, respondents were found to rely on their nuclear families for support. A study in Ghana by Lartey et al. [32] also found that midwives and nurses identified social support from friends and family to help them cope with emotional stress. However, although support from persons in and outside midwives’ workplaces are beneficial to midwives’ coping abilities, it is important that midwives are educated on the extent of information they are legally and ethically allowed to disclose to persons outside their work teams or workplaces to protect clients’ confidentiality.

Midwives in the study mentioned their motivation to pray as an important coping strategy that they adopt when facing challenging cases. To the midwives in this study faith and prayers to seek divine intervention helped them nurture their self-confidence to cope with the challenges that they faced, thereby reducing negative outcomes. Prayers as a coping strategy has also been identified in other similar studies [32].

## Conclusion

In their efforts to provide quality care to women and neonates, Ghanaian midwives face myriad barriers that engenders personal and professional consequences. They are, however, able to adopt coping strategies that enable them to complete their duties. The coping strategies that the midwives adopted stems from their motivation which was found to be underpinned by a strong desire to save the lives of women and neonates and their strong affection for the midwifery profession. The midwives’ motivation helped them to cope with the barriers that they face and spurred them to adopt other coping strategies such as improvising, being preemptive and proactive in the care process and maintaining a support network. Although the midwives are getting positive outcomes for women and neonates, it is vital to investigate further, the long-term effects of some of their coping strategies on the quality of the care that they provide. Improved provision of caregiving resources in Ghanaian maternity services would go a long way to reduce the barriers that midwives face in their work and improve quality care. Pre-and post-registration programs of midwifery education should also focus on developing effective resilience and context relevant coping skills.

## Supplementary Information

**Additional file 1.** INTERVIEW GUIDE.

## Data Availability

The data from which the results of this study emanated are available from the corresponding author on reasonable request.

## Abbreviations

GHS: Ghana Health Service
GT: Grounded Theory
LMICS: Low and Middle-Income Countries
