# Supplementary material for: Midwives’ strategies for coping with barriers to providing quality maternal and neonatal care: a Glaserian grounded theory study
Source: BMC Health Serv Res. 2021 Nov 3;21:1190. doi: 10.1186/s12913-021-07049-0 (PMC8565049; doi:10.1186/s12913-021-07049-0)
Supplement: Supplementary file 1 — Additional file 1. INTERVIEW GUIDE. [file 12913_2021_7049_MOESM1_ESM.docx]

## INTERVIEW GUIDE

**Factors Affecting Ghanaian midwives’ ability to provide quality midwifery care: A Glaserian Grounded Theory study**

**Interview guide**

Name……………………………………..

Facility……………………………………

Date………………………………………. District………………………

Age………………………………………. Gender………………………….

Rank…………………………………….... Years of experience……………. Religion………………………………….. Midwifery route………………. Years of training………………………….

Time start…………………........................ Time end…………………….

1. What is your main motivation to becoming a midwife?
2. Where have you worked/ where was your last station?
3. What is the main difference between working here and working in your old station? What do you find interesting? What do you find difficult/challenging?
4. What are the barriers to your ability to provide quality midwifery care to women and newborns?
5. Can you explain to me how these difficulties affect your ability to provide quality care?
6. Can you describe some instances where these factors have served as barriers in your work?
7. What do you do to reduce the effects of these barriers? (individual, collective, state)
8. What are the effects of these barriers on you as an individual?
9. Can you explain to me how these difficulties affect you as an individual?
10. Can you describe some instances when these difficulties have had effects on you as an individual?
11. What do you do as a person to reduce these effects?
12. What do you think are the main causes of these barriers?
13. Can you describe how these factors cause these barriers?
14. What do you do to be able to carry out your work in the face of the difficulties that you face?
15. Can you give me examples of how you apply or carry out these actions?
16. How effective are these actions in the reduction of the difficulties?
17. What do you think should be done to help midwives to improve quality care?
18. Is there anything else that I have not talked about that you would like to share with me?
